# Therapeutic Lowering of C-Reactive Protein

**DOI:** 10.3389/fimmu.2020.619564

**Published:** 2021-01-29

**Authors:** Rachel V. Jimenez, Alexander J. Szalai

**Affiliations:** ^1^ Department of Immunology, H. Lee Moffitt Cancer Center & Research Institute, Tampa, FL, United States; ^2^ Division of Clinical Immunology & Rheumatology, Department of Medicine, The University of Alabama at Birmingham, Birmingham, AL, United States

**Keywords:** pentraxin, antisense oligonucleotide, autoimmunity, cancer, cardiovascular

## Abstract

In the blood of healthy individuals C-reactive protein (CRP) is typically quite scarce, whereas its blood concentration can rise robustly and rapidly in response to tissue damage and inflammation associated with trauma and infectious and non-infectious diseases. Consequently, CRP plasma or serum levels are routinely monitored in inpatients to gauge the severity of their initial illness and injury and their subsequent response to therapy and return to health. Its clinical utility as a faithful barometer of inflammation notwithstanding, it is often wrongly concluded that the biological actions of CRP (whatever they may be) are manifested only when blood CRP is elevated. In fact over the last decades, studies done in humans and animals (e.g. human CRP transgenic and CRP knockout mice) have shown that CRP is an important mediator of biological activities even in the absence of significant blood elevation, i.e. even at baseline levels. In this review we briefly recap the history of CRP, including a description of its discovery, early clinical use, and biosynthesis at baseline and during the acute phase response. Next we overview evidence that we and others have generated using animal models of arthritis, neointimal hyperplasia, and acute kidney injury that baseline CRP exerts important biological effects. In closing we discuss the possibility that therapeutic lowering of baseline CRP might be a useful way to treat certain diseases, including cancer.

## Preface

This article was written for *Frontiers in Immunology*’s research topic *Diagnostic and Therapeutic Applications of Pentraxin and Pentraxin-Associated Proteins*. Our goal was to review the available evidence - generated by us and by others – that supports targeting of CRP as a therapeutic approach for the treatment of human diseases including cancer. To provide what we think is necessary context we include a brief historical overview of CRP and an update on our understanding of CRP’s general biology and its mode of regulation, and we briefly summarize the use of CRP measurements in clinical practice (although in full disclosure neither of the authors is a clinician with direct experience in that realm). We have done our best to be non-partisan, fair, complete, and balanced, and to give credit to researchers in the field where we think credit is due. Finally, since this is a review paper of limited length we were necessarily judicious and have not referenced the entirety of the excellent work done by all the experts in the field. Those caveats notwithstanding, we sincerely hope that what we have written here stimulates in the reader an appetite for more information about CRP. To feed that appetite we encourage the readers to devour one or more of the many excellent reviews already written by other experts (1–12).

## Historical Overview of C-Reactive Protein

### C-Reactive Protein Gained Acceptance as a Non-Specific Biomarker of Disease Soon After Its Initial Discovery

C-reactive protein (CRP) was discovered during the seminal studies of pneumococcal pneumonia being performed in the laboratory of Oswald T. Avery (1877-1955). There in 1930 in the blood of pneumonia patients, William S. Tillett (1892-1974) and Thomas Francis, Jr. (1900-1969) identified high titers of a substance that was reactive with (and could precipitate from solution) the carbohydrate-rich “C fraction” of *S. pneumonia* (i.e. the fraction containing pneumococcal C-polysaccharide) (13). Today we call this precipitating substance C-reactive protein or CRP. Tillett and Francis also observed the drastic decline and eventual disappearance of the precipitin from patient’s sera coincident with resolution of their febrile period – the first report of CRP’s characteristic rise and fall during what later became known as the “acute phase response” (APR). At the time, to detect CRP Tillett and Francis and their contemporaries relied on a flocculation reaction, wherein CRP in the serum reacted with C-polysaccharide in solution to form a visible precipitate. This assay requires high amounts of CRP and consequently, CRP could only be detected in the serum of patients experiencing severe disease; thus at the time CRP was thought to be absent from the serum of healthy people. Soon thereafter it was recognized that monitoring CRP blood levels might have clinical utility, and this was solidly established by a trio of papers published by Avery’s group in 1941 (14–16). Still today elevation of blood CRP is recognized as a useful “biomarker of inflammation”, and so CRP blood levels are incorporated into numerous disease assessment guidelines. With the eventual development of more accurate and more sensitive techniques for its measurement, CRP was found to be present in normal serum too (17–19) and the first rigorous study of normal CRP values soon followed (20). These findings challenged the earlier *status quo* that CRP was a precipitin present only during infection and raised the possibility that CRP plays a role in normal homeostasis. Recognizing that assays of CRP were of increasing clinical diagnostic value, in 1987 the World Health Organization spearheaded the development of an International Standard for Human CRP. That standard (coded 85/506), comprised of an extract of freeze-dried pooled human serum to which a quantity of human CRP has been added, is stable and is available in ampoules and was shown to be suitable for use as a standard in seven different immunoassays [(21), pp. 21-22]. Preparation 85/506 was meant to be the gold standard against which all national and international secondary CRP standards are meant to be calibrated.

Blood CRP levels in populations of ostensibly healthy people have a positively skewed log-normal distribution, and baseline blood CRP levels in individuals can vary with age, sex, race, genetics, and obesity (22–25). Consequently, even in people whose CRP levels fall within the reference ranges first established by Shine et al. (20) (median 0.8 mg/L with 90% under 3.0 mg/L and 99% under 10 mg/L), CRP values can still vary widely from one healthy person to another. The range of values deemed normal for a physiologic measurement in healthy persons is further complicated by the fact that blood CRP concentrations between 3 and 10 mg/L are considered by some to be indicative of ‘low-grade’ inflammation, i.e. mild inflammation resulting from a variety of persistent metabolic stresses (e.g. atherosclerosis, obesity, obstructive sleep apnea, insulin resistance, hypertension, type 2 diabetes, etc. (4). There is similar wide variability in CRP levels among those whose values are above the normal range; with levels greater than 10 mg/L generally considered to indicate clinically significant inflammation (i.e. the type accompanied by *rubor, calor, dolor*, and *tumor*) and levels above 100 mg/L considered indicative of infection. As mentioned above baseline CRP values are now included in a variety of clinical guidelines for disease assessment. Perhaps the best known example of this is the reference ranges for serum CRP now articulated by the Centers for Disease Control and Prevention and the American Heart Association to estimate cardiovascular risk in otherwise healthy individuals: low-, average-, and high-risk values defined as < 10, 10 – 30, and > 30 mg/L (26). Indeed according to some reports, high baseline CRP is a more robust predictor of future adverse coronary events than is dyslipidemia (27, 28). From cardiovascular disease to cancer to acute kidney injury high CRP levels have been shown to correlate with worse prognosis, and recent evidence indicates CRP is a very good predictor of adverse outcomes for COVID-19 patients (29). Interestingly, animal studies indicate that CRP’s association with autoimmune diseases might be reversed, i.e. the onset of multiple sclerosis, arthritis, and lupus, is delayed in CRP transgenic mice (3, 30, 31). Whether higher baseline CRP predicts later onset of autoimmunity in patients remains to be ascertained. CRP thus has a long history and its elevation in the blood above the normal range has well-established utility as a biomarker of inflammation and disease. As will be discussed later, emerging new evidence indicates that blood CRP can also actively participate in physiological processes when it is in the range deemed normal.

### The Advent of Molecular Biology Ushered in a Deeper Understanding of C-Reactive Protein Structure, Regulation, and Biological Activity

**Figure 1:**
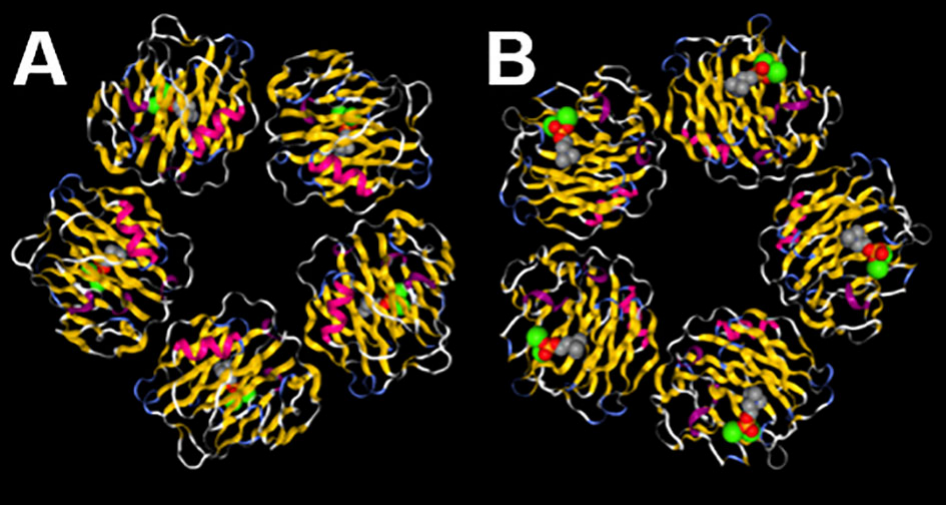
C-reactive protein (CRP) is a pentamer with two distinct faces. **(A)** On the A face each CRP monomer displays an α-helical stretch of residues (fuchsia) adjacent to the C1q and FcR binding sites and on the opposite B face **(B)** each monomer displays residues that help coordinate two Ca^2+^ ions (green spheres) that assist with binding to ligands (phosphocholine shown in gray, red, and orange spheres). The image is a reproduction of structure 1B09 as reported by (35, 36) and was created using NGL (37) accessed on PDB (38–40).

Not only is CRP the prototypical human acute phase protein (32, 33) it is also the prototypical pentraxin (34), an evolutionarily highly conserved class of pattern recognition molecules with the same or highly similar primary, secondary, tertiary, and quaternary structures; human CRP being composed of five globular monomers of 206 amino acids each, non-covalently bound together into a planar ring with a central pore (**Figure 1**) (34, 35, 41). As mentioned earlier the first known biological activity of CRP identified was its ability to bind to and precipitate with the pneumococcal C-polysaccharide (13). This action was later found to rely on CRP binding to phosphocholine (a major constituent of C-polysaccharide) in a Ca^2+^-dependent manner (42–44). While C-polysaccharide/phosphocholine is the canonical and most well-studied ligand for CRP, it is likely not the only ligand to which CRP can bind. For example numerous different groups have reported that CRP also binds to apoptotic cell membranes (45), to various nuclear antigens (46–48), to the oxidized LDL receptor (49), and to many other ligands (9, 12, 50, 51), although some of these reactivity’s have been disputed (52). Additionally, CRP is known to activate the classical pathway of complement by binding and thereby activating C1q (53–55), and to activate numerous myeloid, lymphoid, and endothelial cell responses by binding to a variety of Fc receptors (10, 56). In general it is most likely that by binding one or more of these ligands, by activating complement, and by engaging Fc receptors that CRP mediates its well-described protective influences against bacterial infection (57–61). As will be detailed later, these varied ligand and receptor interactions also support CRP’s participation in various non-infectious diseases. Insofar as is known, the CRP’s of different species share the same or similar ligand binding profiles as human CRP (12).

**Figure 2:**
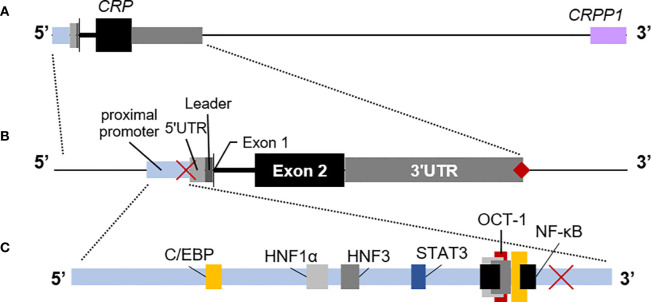
The human C-reactive protein (*CRP*) gene. **(A)** Downstream of *CRP* is the CRP pseudo-gene (*CRPP1*, lilac box), that is likely involved in *cis*-acting regulation of basal *CRP* expression. **(B)** The proximal promoter of *CRP* (light blue box) is typically mapped as the region ~300 nucleotides upstream of the transcription start site, including the TATA binding site at −29 to −26 (red **×**). The short 5’ untranslated region (5’UTR; light gray box) precedes the coding sequence for the 18 amino acid-long leader peptide (dark gray box). Exon 1 encodes the first three amino acids of mature CRP (thin black vertical line) and is immediately followed by the intron. The remainder of CRP is encoded by exon 2 (black box). *CRP* has a long 3’UTR sequence (light gray box) with a poly(A) signal (♦). Each region is drawn approximately to scale. **(C)** The relative locations in the *CRP* promoter of binding elements for hepatic nuclear factor (HNF) 1α and HNF3 that contribute to regulation of constitutive expression of *CRP* (gray boxes) and for C/EBP (yellow boxes), NF-κB (black boxes), and STAT3 (blue box) that contribute to regulation of expression of *CRP* during the acute phase response. Note that C/EBP and NF-κB and the repressor OCT-1 (red box) utilize overlapping elements.

Seminal studies conducted using both rabbit and human cells showed that the hepatocyte is the main site of CRP synthesis and assembly (62, 63). That finding and the subsequent mapping of the human *CRP* gene to chromosome 1 (64) led to more detailed studies of the control of *CRP* expression. While a few studies have reported that some pre-assembled CRP is kept in intracellular pools – from which it is expeditiously released during the APR (65, 66) – the overwhelming evidence from human cells and transgenic mice (67) has solidly established that *CRP* expression – and thus CRP blood levels - is controlled mainly at the transcriptional level. Today, transcriptional regulation of *CRP* is known to be coordinated by a host of cytokines and hepatic transcription factors, with IL-6 and IL-1β being the main cytokines regulating CRP blood levels during inflammatory episodes (68–70). The latter have been shown to bind to various overlapping transcription factor binding elements in the *CRP* promoter, in a region proximal to its coding sequence (**Figure 2**). It is now certain that the levels of blood CRP in healthy individuals is influenced by genetic variation in this promoter region (6, 24), but it is still uncertain whether genetic variation in these or other regulatory elements contributes to differences in the ability to upregulate *CRP* during inflammation. Notwithstanding the latter gap in knowledge, the available evidence indicates that in the absence of inflammation baseline *CRP* expression can be maintained by the transcription factors hepatic nuclear factor (HNF) 1 alpha (HNF1α) and 3 (HNF3) (69). In contrast during inflammation, the increased availability of IL-6 and/or IL-1β supports increased production of the transcription factors C/EBP, STAT3, cFos, and NF-κB, which act synergistically with the *CRP* promoter-bound HNFs to drive high levels of *CRP* transcription (68, 69, 71–73). There is also some evidence that the proximal promoter of *CRP*, perhaps in conjunction with the *CRP* 3’UTR and the downstream pseudogene *CRPP1* can assemble into an enhanceosome (**Figure 2**) that favors more prolonged transcription of *CRP* (67, 74–76). Thus it is most likely that during an APR, the increased quantity and variety of transcription factors available (coupled with their increased nuclear residency?) supports more efficient transcription of *CRP*, thus accounting for the dramatic increase in circulating CRP levels (from ~1 mg/L to upwards of ~200 mg/L) that can be seen within hours during the acute phase response. Presumably when inflammation is resolved and the availability of cytokines and transcription factors is lowered, transcriptional control of *CRP* is handed back to HNFs (69).

## Targeted Lowering of C-Reactive Protein

### Blood C-Reactive Protein Lowering as a Therapeutic for Cardiovascular Disease?

The association of CRP blood levels with cardiovascular disease (CVD) has been of keen interest for many decades, and today baseline CRP is recognized both as an independent marker and predictor of myocardial infarction (MI), stroke, and death from coronary heart disease in ostensibly healthy people. Conclusive evidence for CRP causality in human CVD is still lacking [for more insight see the comprehensive critical reviews by (5, 7)] and the entire issue remains hotly debated, but the results of at least four different clinical trials suggest a role for CRP in the atherogenic process (27, 77–79). Also there is indirect evidence from many different groups suggesting that CRP is causally related to CVD. For example CRP is detected in human atherosclerotic lesions (80–84) and it can activate human endothelial cells (82, 85, 86). The evidence from transgenic animals is equally confusing, with some studies (including some of our own) indicating human CRP might be pathogenic in CVD (87–90) whereas others indicating it is not (91, 92). While the exact contribution of CRP to human CVD remains equivocal and the debate still rages (93), the current authors will not be surprised if future placebo-controlled double-blind studies showed that CRP contributes to the pathophysiology of human CVD.

Causal or not, it has long been accepted that elevated CRP marks the presence of disease. If in certain cases elevated CRP worsens disease, then lowering CRP levels might lead to improvement. CRP lowering can be achieved by lifestyle changes (94) or by targeting inflammatory cytokines [e.g. see (28, 95)], but until recently there was no way to directly and selectively target CRP. This was the major impediment to clinical trials designed to directly test the possible benefits of CRP lowering. Over the last decades several groups have developed approaches to overcome this nagging problem. For example Pepys’ group developed a small molecule inhibitor of CRP (1,6-bis(phosphocholine)-hexane) that they tested in a preclinical rat model (96). The compound works by crosslinking two CRP molecules, thereby blocking its ability to bind endogenous ligands while increasing its clearance from the blood. In rats that had received injections of human CRP prior to ligation of their coronary arteries, the signs of subsequent MI were worsened, indicating that human CRP exacerbates MI. Co-administration of human CRP plus 1,6-bis(phosphocholine)-hexane ablated the exacerbating influence of human CRP (96). This study underscored for the first time the promise of inhibiting CRP as a new approach for cardio-protection in acute MI. Our own group also recently tested a different method of CRP lowering that relies on an antisense oligonucleotide (ASO) (3). ASOs have been shown to be highly effective at promoting the selective degradation of their target mRNAs and to have minimal off-target effects, and several ASOs have already been approved by the United States Food and Drug Administration for use in a variety of disease settings (97, 98). Since CRP is synthesized in the liver and ASOs have a propensity to accumulate in the liver (99), we designed ASOs to selectively target *CRP* mRNA and thereby efficiently reduce blood CRP. We showed that a human *CRP*-specific ASO was effective at lowering baseline CRP and was well-tolerated in healthy volunteers (100), and subsequently others showed that a human *CRP*-specific ASO attenuated CRP elevation (up to 69% compared to placebo) in humans challenged with endotoxin (101). Using species-specific *CRP*-targeting ASOs we showed the approach was also efficacious in rats and mice subjected to experimentally induced MI (89, 90, 102). Specifically, the rat *CRP*-specific ASO achieved a >60% reduction of rat blood CRP levels and improved their heart function and pathology following MI, and treating human CRP transgenic mice with a human *CRP*-specific ASO reduced blood human CRP by >70%. A third approach to CRP lowering is selective apheresis, which has recently been tested with some success in a small number of patients suffering from MI (103, 104) and with mixed results in patients with COVID-19 (105).

### Blood C-Reactive Protein Lowering as a Therapeutic for Other Diseases?

Using our human CRP transgenic (CRPtg) and CRP knockout (CRP^−/−^) mice we have investigated the contribution of CRP in a wide variety of other diseases and, as in the case of CVD, have often observed a beneficial effect of CRP lowering. For example, in mice subjected to surgery-induced bilateral renal ischemia/reperfusion (I/R) injury, a procedure that faithfully mimics acute kidney injury (AKI) in humans receiving kidney transplants, we showed that CRP contributes to the pathogenesis of AKI. Essentially compared to wild type mice, CRPtg had worse outcomes after renal I/R whereas CRP^−/−^ were relatively resistant (106). Following I/R surgery CRPtg showed more disruption of their renal tubules and they had increased urine albumin and serum creatinine compared to wild type. To our surprise these exacerbating effects of CRP were accompanied by increased renal infiltration of myeloid derived cells with suppressor functions (MDSCs; primarily polymorphonuclear PMN-MDSCs) (106, 107). In contrast in the kidneys of CRP^−/−^ that had undergone renal I/R only a few PMN-MDSCs were found. In other experiments we established that CRP can selectively promote the expansion of MDSCs from mouse bone marrow progenitors and increase their suppressive function (i.e. their ability to inhibit T cell proliferation) (31). We conducted other experiments and found that MDSCs directly impact primary renal tubular epithelial cells (RTEC), i.e. in co-cultures MDSCs impaired the ability of RTECs to cycle through S-phase even in the absence of cell-cell contact (unpublished data). The combined findings suggest that CRP potentiates the expansion of MDSCs during AKI, likely by selectively promoting their expansion from bone marrow progenitors. By suppressing cell cycling, the kidney infiltrating MDSCs initiate a deleterious progression of events: the impairment of RTEC proliferation consequently impedes RTEC recovery and thus the restoration of normal tubular architecture, ultimately setting the stage for maladaptive repair and chronic kidney disease. Importantly, we also showed that treating CRPtg mice with the human *CRP*-specific ASO prior to I/R surgery lowered CRP, drastically reduced renal MDSC infiltration, and alleviated AKI (107). Subsequently we established that CRP also enables human neutrophils to manifest T cell suppressive actions (31). Based on these translational findings we think it is likely that therapeutic lowering of CRP might be of benefit in recipients of kidney transplants. We also tested the influence of CRP lowering therapy in the context of an animal model of rheumatoid arthritis (RA), i.e. collagen-induced arthritis (CIA). Thus Jones et al. (108) showed that development of CIA is delayed in CRPtg and accelerated in CRP^−/−^, suggesting that during onset and development of disease CRP plays a protective role. On the other hand, when CRPtg with established CIA (clinical score of ≥ 2) were treated with the human *CRP*-specific ASO they showed less inflammation and improvement of CIA symptoms (100). The protective effect of CRP during onset of disease might reflect the ability of CRP to impair dendritic cell functions while at the same time promote MDSC suppressive activity (30, 31), culminating in a delayed autoimmune response. Conversely, the detrimental effect of CRP seen during active CIA is likely because of its complement activating potential (1). These observations underscore that if CRP lowering is used as a therapy, the timing of CRP lowering will likely be of paramount importance. Indeed this ‘timing effect’ may be the reason why, despite effective CRP lowering, no improvement in symptoms was observed in patients with active RA treated with a CRP-specific ASO (109).

### Something to Think About: Blood C-Reactive Protein Lowering in Cancer?

Despite the success of immunotherapies (e.g. checkpoint inhibitors) to treat solid tumors, there still is a significant fraction of cancer patients that experience no benefit or only a short remission after immunotherapy. Immunotherapy is based on a two pronged approach: 1) boosting the endogenous T cell anti-tumor response to overcome antigen escape and T cell exhaustion (110) and 2) modulating intra-tumoral MDSCs (111). Population studies have established that high CRP levels associate with increased cancer risk (112–114), increased cancer progression (8), and increased cancer mortality (115, 116), and in many cases CRP has been shown to be an independent prognostic factor for cancer (117–122). Given our finding that CRP reprograms myeloid cells we therefore think that cancer might represent a promising opportunity for beneficial CRP lowering, i.e. reducing blood CRP levels should result in fewer MDSCs and a less immunosuppressive tumor microenvironment, thereby potentiating anti-tumor T cell responses. As discussed earlier, our data showed that CRP is an enhancer of MDSC generation and suppressive function and CRP can also inhibit dendritic cell function; therefore we predict that compared to wild type mice, CRPtg should have more MDSCs and more tumor burden while the inverse should be seen in CRP^−/−^ mice. Indeed in pilot studies (unpublished data) using an E0771 orthotopic breast cancer model, we observed CRP^−/−^ had lower tumor burden and lower frequencies of tumor- and spleen-infiltrating MDSCs compared to CRPtg and wild type. These preliminary observations hint at the possibility that CRP contributes to MDSC generation and their eventual infiltration into tumors. Yet to be explored is whether CRP within the tumor microenvironment impedes the anti-tumor response directly (i.e. by inhibiting T cells per se) or indirectly (i.e. by promoting MDSCs). However, based on our previous observations that the CRP ASO decreased MDSC infiltration into I/R injured kidneys and that tumor challenged CRP^−/−^ mice had decreased intra-tumor MDSCs, we predict that a *CRP*-specific ASO should decrease CRP-driven MDSC infiltration into tumors. Targeted lowering of CRP should simultaneously allow for the maturation of tumor-reactive dendritic cells, which in turn would stimulate tumor-reactive T cell responses.

## Summary

Since its discovery nearly a century ago much has been explored and written about CRP’s association with disease, and consequently CRP’s role as a marker of inflammation is solidly established. That being said, CRP is more than just a marker of inflammation, i.e. it did not evolve nor was it maintained by natural selection to provide a faithful indication that something is amiss with a patient. In the last decades evidence has steadily been building that CRP contributes to the maintenance of health and the propagation of some diseases, and for the first time specific CRP lowering is achievable. Therefore the time has come we think, for the biological activities of CRP and therapeutic lowering of CRP to be brought to the forefront in the clinical care setting. Like any therapy CRP lowering might have some untoward effects (e.g. it might increase the risk of infection), nevertheless the time for a placebo-controlled double-blind case-controlled clinical trial of CRP lowering therapy may have finally arrived.

## Author Contributions

RJ and AS contributed equally to this work. All authors contributed to the article and approved the submitted version.

## Conflict of Interest

The authors declare that the research was conducted in the absence of any commercial or financial relationships that could be construed as a potential conflict of interest.
